# Impact of Five Soy Proteins on Lean Chicken Breast Systems with Varying Moisture Contents: Cooking Loss, Texture, Microstructure, and T_2_ NMR

**DOI:** 10.3390/foods14030427

**Published:** 2025-01-28

**Authors:** Weilun Lin, Shai Barbut

**Affiliations:** Department of Food Science, University of Guelph, Guelph, ON N1G 2W1, Canada

**Keywords:** gel structure, hybrid meat, NMR, soy protein, texture

## Abstract

With increasing global meat consumption, meat–plant hybrid products have gained interest as a sustainable alternative. Soy proteins have been used in small quantities (2–3%) as meat extenders, yet limited data exist on their use at higher levels. Here, five commercial soy proteins (four isolates: SPI-A to -D; one concentrate: SPC) were used for meat replacement in lean meat batters with 0/40/80% added water. Cooking loss, texture, light micrographs, and T_2_ relaxation were analyzed. At 33% and 66% meat replacement, soy protein treatments maintained comparable or reduced cooking loss; SPI-D and SPC were the least and most effective, respectively. Complete replacement eliminated cooking loss in 0% and 40% water systems but failed to form self-supporting gels in the 80% system. At 33% replacement, SPI-A to -C generally increased hardness, whereas increasing the replacement level further to 66% decreased it. In the 0% and 80% systems, SPI-A treatments exhibited hardness comparable to controls, SPI-D treatments drastically reduced hardness, and SPC treatments maintained greater hardness than the controls even at 66% replacement. Micrographs offered potential explanations for these macroscopic measurements. NMR T_2_ data indicated that soy proteins restricted water mobility both pre- and post-cooking. Specifically, in the 40% and 80% systems, the T_22_ peaks (expelled liquid) of the hybrid samples containing 33% SPI-A were ~350 ms and 760 ms, compared to ~570 ms and 1170 ms for the meat controls, respectively. In conclusion, most soy proteins (except SPI-D) enhanced water binding, with SPI-A showing optimal texture and SPC showing promise as a more economical alternative.

## 1. Introduction

The world population is projected to reach nine billion by 2038 [1]. Rising meat consumption inevitably follows global population growth and increased household income, particularly in Latin American and Asian countries [2]. This elevated meat demand can strain the current meat production systems, exacerbating animal welfare concerns and environmental issues within the already compromised ecosystem.

As sustainability becomes a growing concern in today’s society, flexitarian diets have emerged as a popular trend. As a result, hybrid meat products are gaining attention in both the meat industry and academia as an intermediate between current meat-oriented and future plant-oriented diets, facilitating this dietary transition alongside the evolution of plant-based meat analogs. Furthermore, hybrid meat production provides a promising and sustainable approach to valorizing otherwise wasted meat materials (i.e., small trims) by restructuring them with plant proteins [3]. Poultry, particularly chicken, is a predominant source of meat consumed worldwide [4]. As a result, the poultry industry generates substantial amounts of small trims during processing, which are often underutilized.

Soybean and its derivatives have long been used (at low concentrations: 2–3%) to improve the functionality and reduce the cost of processed meat products [5]. In general, bean flour contains 20–30% protein, whereas protein concentrates and isolates contain over 65% and 90% protein, respectively. Previously, soy flour [6,7], soy protein concentrate [6,8], and soy protein isolate [9,10,11,12,13] have been used to supplement different meat products. In fact, soy protein preparation methods have advanced over the last two decades. Few, if any, studies have compared multiple contemporary commercial soy proteins or investigated the effects of incorporating elevated levels of soy proteins in lean meat systems with different moisture contents. Hence, the present study aims to fill this gap by assessing how five different commercial soy proteins (four isolates, one concentrate), replacing meat at 0%, 33%, 66%, and 100%, impact the texture and structure of lean chicken meat systems with 0%, 40%, and 80% added water.

## 2. Materials and Methods

### 2.1. Materials

Deboned and skinned chicken breast fillets were purchased from a local wholesaler (Costco Wholesale Corporation, Guelph, ON, Canada). Visible fat and connective tissue were removed, and the meat was diced into approximately 2 × 2 cm pieces, then chopped for 40 s at a low-speed setting in a bowl chopper (Schneidmeister SMK 40, Berlin, Germany). The chopped meat was vacuum-sealed in 0.5 kg portions and kept at −20 °C. The meat protein content was 22.61 ± 0.13%, determined in triplicate using the Dumas combustion method (AOAC 992.15) with a nitrogen-to-protein conversion factor of 6.25. Four soy protein isolates (**SPI-A to -D**; 86.86 ± 0.75% protein content, determined by Dumas) and one soy protein concentrate (**SPC**; 64.07 ± 0.05% protein, determined by Dumas) were manufactured by the Solae Company and supplied by the Hela Spice Company, Uxbridge, ON, Canada.

### 2.2. Sodium Dodecyl Sulfate–Polyacrylamide Gel Electrophoresis (SDS–PAGE)

Reducing SDS–PAGE was performed on the five soy proteins using a Bio-Rad 12% acrylamide precast gel (Bio-Rad, Hercules, CA, USA). Each soy protein powder was solubilized in deionized water to obtain a 1% *w*/*v* protein dispersion. 10 µL solubilized soy protein was diluted with 10 µL deionized water, then mixed with 20 µL reducing sample buffer (19 µL 2× Laemmli buffer, 1 µL β-mercaptoethanol). Sample mixtures were incubated in a 95 °C water bath for 5 min before electrophoresis. 10 µL of each sample and 5 µL of Precision Plus Protein™ All Blue molecular weight standard (Bio-Rad) were loaded onto the gel placed in running buffer (100 mL 10× Tris-Tricine-SDS running buffer, 900 mL deionized water) and electrophoresed at 120 V for approximately 60 min. The gel was stained with Coomassie Blue solution for 60 min, destained overnight, and then imaged using Gel Doc™ EZ Imager (Bio-Rad), as described by Marciniak et al. [14].

### 2.3. Soy Protein Hydration and Hybrid Meat Batter Production

Soy protein powders were hydrated with deionized water to achieve protein levels equivalent to those in each of the three meat batter systems and stored at 4 °C overnight. Subsequently, prehydrated soy proteins were manually mixed (~60 rpm) with 2.5% total sodium chloride (*w*/*w*) for 30 s. Frozen meat was defrosted overnight (at 4 °C). Three meat batter systems with varying added moisture contents were prepared in triplicate following the procedure outlined by Lin and Barbut [15] with slight modifications. The control (**CL**) treatment of each system consisted of meat, deionized water (at 0%, 40%, and 80% of the meat), and 2.5% *w*/*w* total sodium chlorite. Following 30 s of manual mixing, the meat batters were kept at 4 °C for 1 h to allow the extraction of salt-soluble myofibrillar proteins.

Treatments were prepared for each system by substituting 33%, 66%, and 100% (*w*/*w*) of the CL meat batter with each of the five hydrated soy proteins. Each treatment sample (hybrid meat batter: 33% and 66% substitution; soy protein batter: 100% substitution) was hand-mixed (~60 rpm) for 30 s, stuffed into three 50 mL polyethylene test tubes (30 g/tube) and centrifuged (Fisher Scientific, Model 225, Pittsburgh, PA, USA) at the low-speed setting for 40 s to eliminate entrapped air.

### 2.4. Cooking and Cooking Loss

Test tubes containing samples were cooked in a water bath (Haake, SC 100, Newington, CT, USA) from 20 °C to 72 °C (internal temperature). The internal temperature was continuously monitored using a thermocouple (Omega, Model HH23, Stamford, CT, USA). Heated sample tubes were chilled to 24 °C in a water bath set to 20 °C, after which cooking loss was measured by decanting the fluid and calculating the % (*w*/*w*) fluid lost relative to the original sample weight. Measured samples were sealed and kept at 4 °C overnight.

### 2.5. Texture Profile Analysis

Cooked samples (previously at 4 °C) were brought to room temperature. Six pucks (diameter: 19 mm; height: 10 mm) were cut from each sample core for texture profile analysis, using a texture analyzer (TA-XTPlusC, Texture Technologies Corp., Scarsdale, NY, USA) equipped with a 50 kg load cell. Hardness, springiness, cohesiveness, gumminess, chewiness, and resilience parameters were measured using a two-cycle 50% compression with a cylindrical flat probe (diameter: 100 mm) descending at a rate of 1.5 mm/s [16].

### 2.6. Light Microscopy

Cooked samples were cut into 3 mm thick slices, fixed, embedded in paraffin, sectioned (4–7 μm), and stained with hematoxylin–eosin for protein and periodic acid–Schiff for carbohydrate visualizations, as per the procedure outlined by Youssef and Barbut [17]. Light micrographs were obtained using a digital camera (Model DP71, Olympus Optical Ltd.) attached to a light microscope (Model BX60, Olympus Optical Ltd., Tokyo, Japan).

### 2.7. Pulsed NMR T_2_ Relaxometry

T_2_ relaxation times of the raw and cooked samples with 33% meat replacement by hydrated soy proteins were measured for each meat batter system using a 20 MHz (0.47 T) bench-top NMR spectrometer (Bruker Canada, mq 20 series, Milton, ON, Canada) maintained at 5 °C. The measurements followed the method described by Lin and Barbut [15]. Free induction decay (**FID**) data were acquired using a CPMG spin echo pulse train with 16 scans and a 10 s recycle delay between scans. Using the corresponding raw control meat batters, the 90° and 180° pulse lengths were optimized to 9.44 μs and 18.34 μs for the 0% added water system, 9.50 μs and 18.48 μs for the 40% added water system, and 9.58 μs and 18.64 μs for the 80% added water system, respectively. The 90 to 180° pulse separation time (τ) was set to 1.2 ms. The FID data were analyzed using the CONTIN algorithm (Bruker Corp.) to obtain T_2_ relaxation profiles. The relaxation times were determined from the peak positions.

### 2.8. Color Evaluation

From each cooked sample, three 10-mm-thick samples were freshly cut and measured using a colorimeter (Konica-Minolta CR-400 Chroma Meter, Osaka, Japan) with a 2° viewing angle and D65 illuminant setting to assess the lightness (L*), redness (a*), and yellowness (b*) parameters, according to the Commission Internationale de l’Éclairage (CIE) system.

### 2.9. Statistical Analysis

The experiment employed a completely randomized design with three independent replications for each moisture level (0%, 40%, 80%). ANOVA was performed, followed by Tukey’s post hoc multiple comparison test, to determine significant differences among formulations within each moisture level (*p* < 0.05). Statistical analyses were conducted using SAS software 9.4 (SAS Institute Inc., Cary, NC, USA).

## 3. Results and Discussion

### 3.1. SDS PAGE

**Figure 1:**
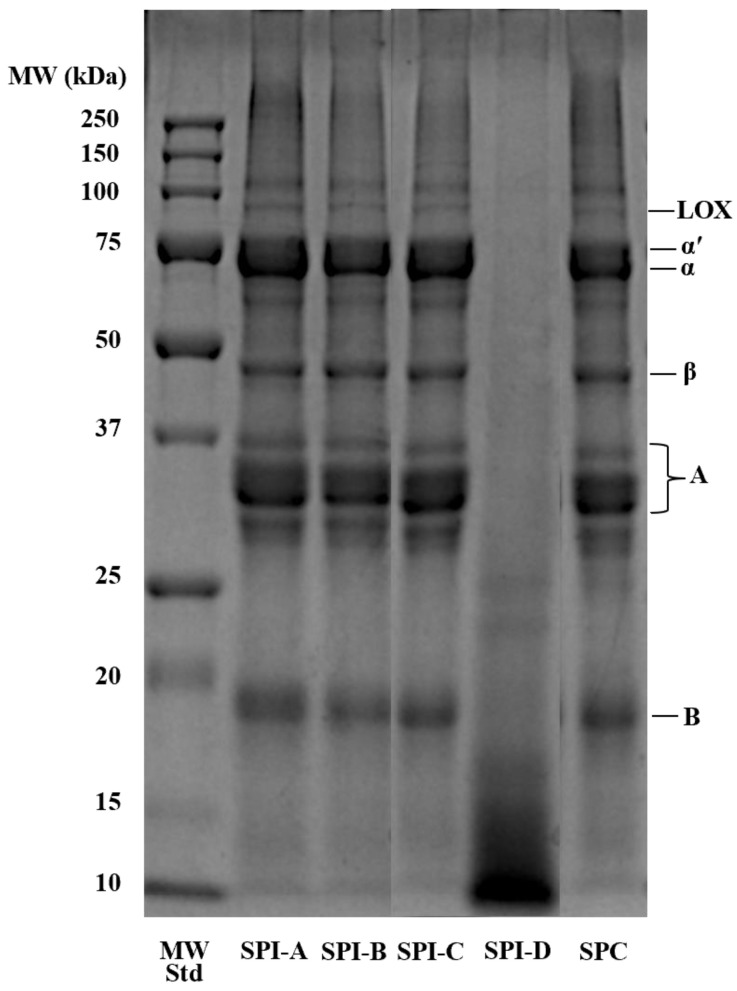
Reducing SDS-PAGE gel of soy protein powders. MW Std: molecular weight standard; SPI-A to -D: soy protein isolates A, B, C, and D; SPC: soy protein concentrate; LOX: lipoxygenases; α′, α, and β: subunits of β-conglycinin (7S); A and B: acidic subunit and basic subunit of glycinin (11S), respectively. (The SPI-C and SPI-D lanes were transposed horizontally from the original image for proper alignment.)

SDS-PAGE was performed to compare the molecular weight profiles of the five soy protein powders, which likely underwent different processing conditions. Processes such as heat treatment and hydrolysis may alter the molecular weight profile of soy protein, including the 7S/11S globulin ratio, which can largely impact soy protein gel strength [18]. Figure 1 shows the electrophoretic patterns of the soy protein subunits. SPI-A to -C and SPC exhibited similar, typical soy protein electrophoretic patterns for the 7S (α′, α, and β subunits of β-conglycinin) and 11S (acidic and basic subunits of glycinin) components [19]. On the other hand, SPI-D exhibited no defined bands above 25 kDa and showed smearing below 20 kDa, suggesting that it may have been hydrolyzed during processing.

### 3.2. Cooking Loss

**Figure 2:**
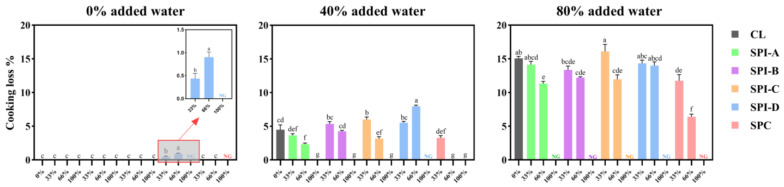
Mean cooking loss values (with standard error bars) of three lean meat batter systems (0%, 40%, and 80% added water) with 33 to 100% meat replacement by soy proteins (as indicated on the x-axis), n = 9. CL: meat control with 0% soy protein; SPI-A to -D: soy protein isolates A, B, C, and D; SPC: soy protein concentrate. NG: no gel formed. Within each system, treatments with different letters (^a–g^) are significantly different (*p* < 0.05).

Overall, within all three systems, the cooking loss of lean meat batters showed no or little improvement when partially substituted with hydrated soy protein powders (Figure 2).

In the 0% added water system, the control (**CL**) and treatments with 33%, 66%, and 100% SPI-A to -C and SPC showed no cooking losses. The incorporation of SPI-D resulted in a subtle but statistically significant cooking loss (less than 1% *w*/*w*) at the 33% and 66% replacement levels and failed to form a self-supporting gel at 100%.

In the 40% system, at 33% replacement, all soy proteins—except for SPI-C—resulted in cooking losses similar to the CL. At 66% replacement, SPI-A, SPI-C, and SPC significantly reduced cooking loss, consistent with findings in previous studies [9,12,13], although those studies focused on the addition of dry soy protein powder. On the other hand, SPI-B had no significant effect, while SPI-D significantly increased cooking loss compared to the CL. At 100% replacement, most soy protein treatments showed no cooking losses, whereas SPI-D resulted in no gel formation and thus no measurable cooking loss.

In the 80% system, the CL had a substantial cooking loss (approximately 15% *w*/*w*). At 33% replacement, all SPI treatments had no significant difference from the CL, while SPC reduced cooking loss by 20%. At 66% replacement, all soy proteins except SPI-D significantly decreased cooking loss compared to CL; however, increasing the replacement level from 33% to 66% did not improve water holding in the SPI-B and SPI-D treatments. At 100% replacement, all the soy proteins failed to form gels.

In summary, these results demonstrate that, in a lean meat system with 40% (and possibly slightly more) added water, a 66% replacement of meat with soy proteins (except for SPI-D; prehydrated to match the protein content of the meat) significantly reduced cooking loss. This finding extends the knowledge in the literature, where soy protein isolates and concentrates were mostly added at low concentrations and as dry powders.

### 3.3. Texture Profile Analysis

#### 3.3.1. Hardness

**Figure 3:**
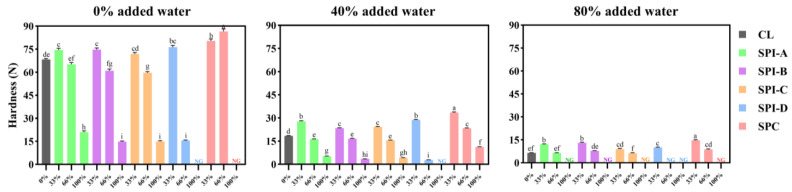
Hardness means (with standard error bars) of three lean meat batter systems (0%, 40%, and 80% added water) with 33 to 100% meat replacement (as indicated on the x-axis) by soy proteins, n = 18. CL: meat control with 0% soy protein; SPI-A to -D: soy protein isolates A, B, C, and D; SPC: soy protein concentrate; NG: no gel formed. Within each system, treatments with different letters (^a–i^) are significantly different (*p* < 0.05).

In the 0% added water batter system (Figure 3), at the 33% substitution level, all the soy proteins significantly increased hardness compared to the CL (68.3 N), with SPC resulting in the most pronounced increase (80.4 N). In contrast, Berry et al. [8] reported that replacing 20% of a ground beef patty with a soy protein isolate or concentrate (each hydrated to match beef protein content at 19%) resulted in lower shear forces than the all-beef patty. This discrepancy may stem from the different types of soy proteins used, the inherently greater toughness of beef than that of chicken and/or differences in meat particle size between the studied systems. When replacing 66% of the meat, SPI-A resulted in a hardness value comparable to the CL, whereas SPI-B to -D significantly reduced hardness; notably, SPI-D decreased hardness by over 75% compared to CL. Conversely, SPC resulted in the highest hardness among all the treatments at this level, reaching 86.5 N. At complete replacement, all SPIs, except for SPI-D (texture parameters not measurable), led to significantly reduced hardness (14–21 N) compared to the CL. On the other hand, SPC formed crumbles that were not cohesive enough to form gels that could be measured.

In the 40% system, 33% replacement by soy proteins significantly increased the hardness compared to CL (18.4 N) in all cases. At 66% replacement, the SPI-A to -C treatments achieved hardness (~16 N) fairly similar to CL; however, SPI-D dramatically decreased the hardness (<3 N), whereas SPC produced the hardest gel at this level. At 100% replacement, all SPIs resulted in hardness values <5 N, whereas the SPC treatment remained the hardest (11.2 N).

In the 80% system, at 33% substitution, all soy proteins significantly increased hardness compared to CL (6.5 N), with SPC resulting in the most pronounced increase. At 66% substitution, the SPI-A to -C treatments exhibited hardness comparable to CL, whereas the SPI-D treatment was too brittle for texture measurement, and the SPC treatment had the highest hardness. At full replacement, none of the soy proteins formed a gel.

Overall, in all three systems, SPI-A to -C had a similar effect on textural hardness across all replacement levels, while SPI-D resulted in a lower hardness than the other soy proteins did at 66% replacement. SPC treatments consistently exhibited a stronger firming effect than the SPIs. This effect might be attributed to the greater quantity of SPC powder (due to its lower protein content than SPIs) required to match the protein level of each meat system.

#### 3.3.2. Other Texture Parameters

Table S1 presents the other texture profile parameters: springiness, cohesiveness, gumminess, and chewiness. Several treatments lack measurable results due to very weak or no gel formation.

Regarding springiness, in the 0% system, at 33% replacement, the SPI treatments exhibited significantly higher values compared to CL, while the SPC treatment showed no significant difference. At 66% replacement, SPI-A to -C further increased springiness, while SPI-D led to significantly lower springiness, and the SPC treatment remained similar to CL. A complete replacement with SPI-A to -C resulted in springiness similar to that at 66% substitution, whereas the springiness of the SPI-D and SPC treatment was not measurable. In the 40% system, soy proteins generally increased springiness to a range of 0.90–0.95 from the CL (0.86). However, the SPI-D treatment was an exception, as its springiness significantly dropped to 0.59. In the 80% system, soy proteins showed the greatest improvement in springiness among all three systems, particularly at the 33% replacement level, although this effect was only slightly less prominent at 66% replacement.

In terms of cohesiveness, in the 0% system, at both the 33% and 66% replacement levels, soy protein treatments (except SPI-D at 66%) were significantly higher than the CL (0.59). However, increasing the SPI-D level from 33% to 66% resulted in a less cohesive texture. At 100% replacement, the SPI-A and SPI-C treatments remained more cohesive (0.70 and 0.69, respectively) than the CL, while the SPI-B (0.54) and SPI-D (0.37) treatments were significantly less cohesive. In the 40% system, a similar pattern with slight deviations was observed: at full substitution, all soy protein treatments (except SPI-D) exhibited cohesiveness comparable to CL. In the 80% system, the SPI-A to -C and SPC (33% and 66% substitution) and SPI-D (33% substitution) treatments showed significantly higher cohesiveness than the CL.

Gumminess and chewiness followed a similar pattern to hardness. The resilience of the cooked samples was significantly increased by replacement with soy proteins in all three systems, except for SPI-B and SPI-D at the 66% replacement level. Overall, the SPI treatments demonstrated greater resilience than the SPC treatment.

### 3.4. Light Microscopy

**Figure 4:**
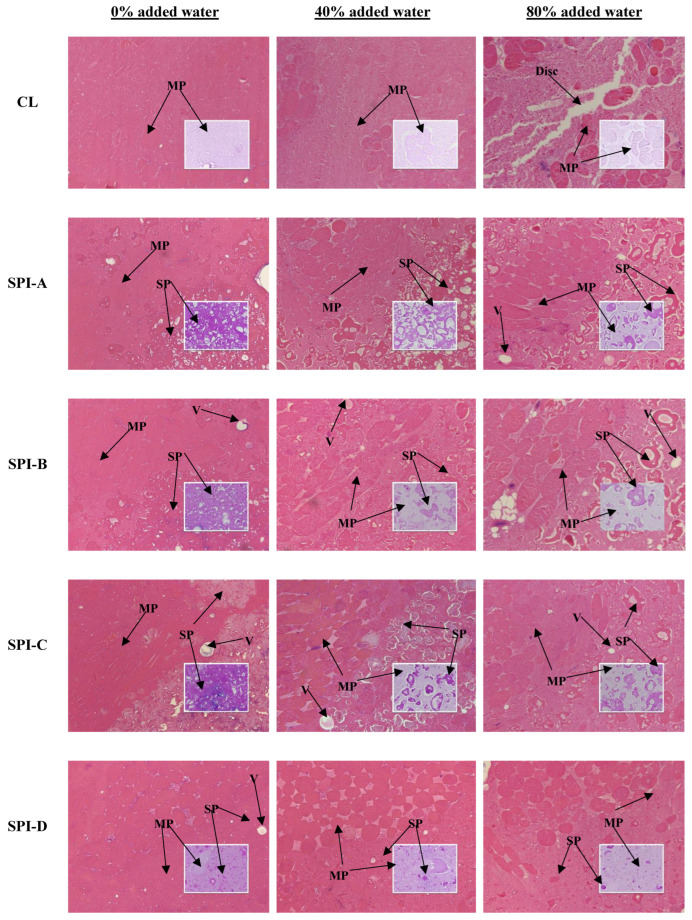

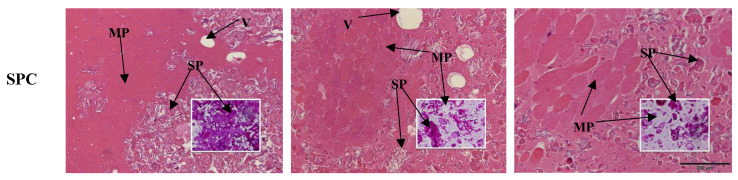
Hematoxylin–eosin-stained light micrographs of three cooked meat batter systems (0%, 40%, and 80% added water) with 33% meat replacement by soy proteins. Periodic acid–Schiff-stained sections are embedded into hematoxylin–eosin images. CL: control; SPI-A to -D: soy protein isolates A, B, C, and D; SPC: soy protein concentrate; Dsic: disconnection; V: void; MP: meat protein. Bar = 200 μm. Magnification: 10×.

Figure 4 shows light micrographs of three lean meat batters (0%, 40%, and 80% added water) containing 33% hydrated soy proteins. In the 0% system, the CL showed a compact matrix with minimal open structures, similar to our previous work [20]. In the soy protein treatments, air bubbles were occasionally entrapped within the meat matrix, which was likely due to increased viscosity as a result of soy protein additions [15]. SPI-A to -C and SPC formed larger aggregates containing some air bubbles, whereas SPI-D remained as smaller particles without forming aggregates. This observation is consistent with the molecular weight profile (Figure 1), which suggests that SPI-D was previously hydrolyzed. Additionally, the SPC aggregates had a more intense purple hue (i.e., retained more PAS stain) compared to those of the SPIs; this can be explained by its higher carbohydrate content.

In the 40% system, the aggregates of soy proteins were generally less dense compared to those in the 0% system. SPI-A appeared more interconnected and porous than the other soy proteins, suggesting that SPI-A formed a more cohesive gel matrix (Table S1; although not statistically significant) that also appeared to retain more air bubbles. Like in the 0% system, SPI-D was loosely dispersed within the meat matrix. It is speculated that at higher inclusion levels, the smaller particles of SPI-D, with greater surface area and no aggregate structure, may interfere with the meat batter’s structure. This could explain why, at 66% replacement, SPI-D resulted in the lowest hardness and highest cooking loss among all the soy proteins (Figure 3).

In the 80% system, the CL batter exhibited large disconnections, indicating disruptions of the meat gel matrix. At 33% substitution, soy proteins mitigated the matrix disruption observed in CL, which also resulted in more rigid structures in the hybrid meat batters; however, this improvement was not necessarily reflected in the cooking loss of the soy protein treatments. SPI-A to -C showed somewhat similar aggregate structures, with some air bubbles confined within them.

Overall, the distribution of hydrated soy proteins within the meat matrix became increasingly sparse as the moisture level increased.

### 3.5. NMR T_2_ Relaxometry

**Figure 5:**
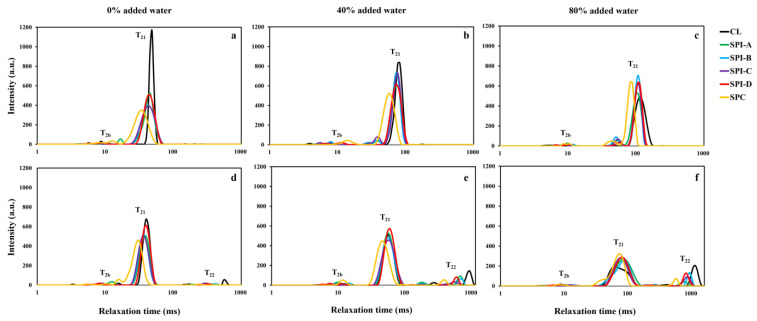
NMR T_2_ relaxation profiles of three lean meat batter systems (0%, 40%, and 80% added water) with 33% meat replacement by soy proteins. CL: meat control with 0% soy protein; SPI-A to -D: soy protein isolates A, B, C, and D; SPC: soy protein concentrate. Panels (**a**,**d**): raw and cooked batters with 0% added water; panels (**b**,**e**): 40% added water; panels (**c**,**f**): 80% added water. Each data point represents an average of at least three replications. T_2b_, T_21_, and T_22_: distinct T_2_ peak regions.

NMR T_2_ relaxation profiles of three lean meat batters (0%, 40%, and 80% added water) containing 33% hydrated soy proteins are displayed in Figure 5. Pulsed NMR T_2_ relaxation was employed to investigate water mobility in the three meat systems with partial meat replacement by hydrated soy proteins. Peak regions were identified and associated with three water components in the muscle structure: T_2b_ (5–15 ms), T_21_ (30–95 ms), and T_22_ (>100 ms), corresponding to water tightly bound to charged protein molecules, water immobilized within the protein matrix, and water in the extramyofibrillar space, respectively [15,21]. Specifically, in this study, T_21_ provided insight into how hydrated soy proteins can alter water mobility within the protein gel matrix. In general, within the same peak region, a shorter T_2_ time indicates more restricted water mobility.

In the raw, 0% added water system, the CL batter had a T_21_ time of ~49 ms. At 33% replacement, soy proteins decreased the T_21_ relaxation time of the CL in all cases. The T_21_ time was ~43 ms for the SPIs and ~36 ms for the SPC treatments. T_21_ values in the cooked state followed a similar trend to that in the raw state. After cooking, the T_21_ values of the SPI-A to -C treatments were approximately 38 ms, whereas the T_21_ time of the SPI-D treatment fell in the same range as the CL (~41 ms), indicating that SPI-D did not improve the water binding of the cooked meat batter. However, it should be noted that a minor T_22_ peak was found in the CL batter at ~606 ms. This phenomenon is consistent with a previous study reporting that hydrated soy protein did not significantly alter the T_21_ time but reduced the T_22_ time in a hybrid meat emulsion [22]. In the present study, T_2_ relaxation profiles did not fully align with the cooking loss results, as the SPI-D treatment led to cooking loss (Figure 2), while the CL and other soy proteins did not. On the other hand, the SPC treatment exhibited the best water binding (~31 ms), which can be attributed to the greater quantity of SPC powder (relative to the water used for hydration) required to compensate for its lower protein concentration compared to the SPI treatments.

In the raw, 40% system, the CL had a T_21_ time of ~84 ms, whereas the SPI treatments had T_21_ values of ~75 ms, and the SPC treatment had a T_21_ time of ~60 ms. In any case, soy proteins effectively helped retain water in the protein matrix, even prior to cooking. In the cooked samples, the T_21_ values for SPI-A to -C and CL were approximately 54 ms, with T_22_ peaks ranging from 531 to 962 ms. The SPI-D treatment exhibited the longest T_21_ time (~60 ms), suggesting that water molecules were more mobile in the SPI-D samples, with a greater tendency to migrate out of their protein matrix. The SPC treatment had a T_21_ time of ~49 ms and a T_22_ time of ~382 ms. Although the T_21_ ranking pattern does not fully align with cooking loss, it may explain the significant increase in cooking loss observed with the SPI-D treatment when the meat replacement level increased from 33% to 66% (Figure 2).

In the raw, 80% system, all the soy proteins reduced the T_21_ time compared to CL (~117 ms), with all SPI treatments at ~109 ms and the SPC treatment at ~90 ms. However, once cooked, the CL exhibited a T_21_ peak at ~64 ms and a T_22_ peak at ~1172 ms. The CL’s T_21_ was shorter than that of all the soy protein treatments, but its T_22_ was the longest. Additionally, CL had the largest T_22_ peak area, indicating the migration of immobilized water (T_21_) from the meat protein matrix to the extramyofibrillar space (T_22_). This explains the shorter T_21_ time in the cooked CL sample compared to the soy protein treatments. SPI treatments generally had T_21_ and T_22_ times comparable to that of CL, whereas SPC showed the shortest T_21_ and T_22_ values among all, consistent with their cooking loss at the 33% substitution level.

Overall, although the T_2_ relaxation profiles did not always fully align with the cooking loss results, they provide insight into the molecular level of water binding. Also, it is important to note that T_2_ measurements were taken at 5 °C, while cooking loss was measured at 24 °C.

### 3.6. Color Parameters

Substitutions with soy protein significantly reduced the lightness (L*) values of the cooked samples in all three systems, regardless of the choice and replacement level of soy protein (Table 1). L* values exhibited a decreasing trend with increasing levels of meat replacement. Similar results were reported by Lin and Barbut [15] when other plant proteins were added to a lean chicken meat batter. SPI-D led to the smallest decrease in L*, while SPI-A resulted in the largest reduction, with SPI-B, SPI-C, and SPC showing intermediate effects. Additionally, there was a pattern indicating that as the additional moisture content increased (from 0% to 80% added water), the overall difference in lightness between the CL and soy protein treatments diminished. At the 33% and 66% replacement levels, the yellowness (b*) value generally decreased in the SPI-A, B, and C treatments but it increased in the SPI-D and SPC treatments. The differences in L* and b* among soy protein treatments can be attributed to their intrinsic color differences (Figure S1). In terms of redness (a*), the substitution with soy proteins had a subtle, though statistically significant, effect on the meat batters across the three systems. However, such a difference was not noticeable to the naked eye.

## 4. Conclusions

This study examined the effects of partial and full meat replacement with four soy protein isolates and one soy protein concentrate (hydrated to match meat batter protein contents) on the physical and structural properties of lean meat batter systems with varying added moisture levels. Replacing 33% of meat batters with hydrated soy proteins generally resulted in cooking loss comparable to that of meat-only batters. At 66% replacement, cooking loss decreased further, particularly with SPC, except for SPI-D, which was the least effective at retaining moisture. Complete replacement resulted in no cooking losses in the 0% and 40% added water systems for most soy proteins, except SPI-D; none formed stable gels in the 80% system.

In terms of textural hardness, 33% replacement with SPI-A to -C generally increased the hardness of hybrid meat batters compared to CL, while 66% replacement reduced hardness in most cases. With 66% SPI-A, the hybrid meat batter was able to match the hardness of control meat batters containing 0% and 80% added water. On the other hand, SPI-D caused significant decreases in hardness (over fourfold in the 0% and ninefold in the 40% added water systems) when the substitution ratio increased from 33% to 66%. SPC treatments showed a similar pattern to the SPI-A to -C treatments in all systems but remained harder than CL even at 66% replacement.

Light micrographs offered potential explanations for the water-holding and textural hardness patterns in meat batters partially substituted with different soy proteins. SPI-D exhibited the smallest particles across all three meat matrices, aligning with its molecular weight profile (<25 kDa bands). Together, this evidence suggests that SPI-D underwent hydrolysis during manufacturing. NMR T_2_ profiles provided valuable insights into water mobility within the three moisture systems and offered a complementary explanation for the cooking loss patterns observed through the microstructure.

Overall, the SPIs, particularly SPI-A, demonstrated a comparable texture and superior water-binding ability to those of the control meat batters. Conversely, under the current hydration conditions, SPC resulted in much higher textural hardness and lower cooking loss than the control, as it requires less hydration to match the meat batter’s protein level due to its inherently lower protein content compared to SPIs. Nonetheless, SPC shows promise as a more economical option for hybrid meat batters, with further research needed to optimize hydration conditions and replacement levels to reverse-engineer the all-meat texture.

## Figures and Tables

**Table 1:** Mean color parameters (L* = lightness; a* = redness; b* = yellowness; with standard error) of cooked samples in three meat batter systems (0%, 40%, and 80% added water) with 33 to 100% meat replacement by soy proteins. n = 9.

		0% Added Water			40% Added Water			80% Added Water		
Treatment	Replacement (%)	L*	a*	b*	L*	a*	b*	L*	a*	b*
CL	0	77.9 ± 0.38 ^a^	2.1 ± 0.08 ^bc^	12.8 ± 0.10 ^c^	78.2 ± 0.11 ^a^	1.0 ± 0.03 ^ab^	11.4 ± 0.15 ^cd^	78.3 ± 0.26 ^a^	0.5 ± 0.03 ^a^	10.3 ± 0.13 ^bc^
SPI-A	33	69.1 ± 0.25 ^g^	1.7 ± 0.11 ^d^	10.5 ± 0.21 ^ef^	72.5 ± 0.13 ^e^	0.2 ± 0.08 ^de^	10.4 ± 0.09 ^ef^	73.2 ± 0.19 ^d^	−0.2 ± 0.10 ^bc^	9.9 ± 0.17 ^cd^
	66	62.8 ± 0.15 ^i^	−0.2 ± 0.07 ^h^	10.3 ± 0.19 ^fg^	65.9 ± 0.21 ^g^	−0.9 ± 0.04 ^f^	10.2 ± 0.09 ^fg^	65.6 ± 0.17 ^f^	−1.3 ± 0.04 ^d^	9.7 ± 0.11 ^cde^
	100	55.1 ± 0.24 ^k^	0.5 ± 0.05 ^g^	10.9 ± 0.23 ^e^	56.9 ± 0.21 ^i^	−0.7 ± 0.02 ^f^	8.5 ± 0.06 ^h^	NG	NG	NG
SPI-B	33	73.3 ± 0.40 ^bc^	1.8 ± 0.03 ^cd^	10.9 ± 0.11 ^e^	75.5 ± 0.14 ^b^	0.8 ± 0.09 ^bc^	10.0 ± 0.13 ^fg^	75.7 ± 0.19 ^b^	−0.1 ± 0.09 ^b^	9.2 ± 0.15 ^ef^
	66	71.3 ± 0.29 ^ef^	0.0 ± 0.05 ^h^	10.6 ± 0.08 ^ef^	72.6 ± 0.21 ^e^	−0.6 ± 0.04 ^f^	9.7 ± 0.14 ^g^	72.4 ± 0.15 ^d^	−0.9 ± 0.06 ^d^	8.8 ± 0.12 ^f^
	100	67.0 ± 0.12 ^h^	−0.3 ± 0.05 ^h^	9.8 ± 0.09 ^g^	69.4 ± 0.15 ^f^	−0.6 ± 0.02 ^f^	8.8 ± 0.13 ^h^	NG	NG	NG
SPI-C	33	72.0 ± 0.27 ^de^	0.9 ± 0.09 ^f^	10.8 ± 0.06 ^ef^	74.2 ± 0.22 ^cd^	0.0 ± 0.13 ^e^	9.9 ± 0.11 ^fg^	74.6 ± 0.13 ^c^	−0.6 ± 0.14 ^c^	9.4 ± 0.17 ^def^
	66	66.8 ± 0.28 ^h^	−1.2 ± 0.11 ^i^	9.6 ± 0.09 ^gh^	69.2 ± 0.23 ^f^	−1.8 ± 0.12 ^g^	8.8 ± 0.10 ^h^	69.3 ± 0.35 ^e^	−2.1 ± 0.06 ^e^	8.0 ± 0.14 ^g^
	100	60.6 ± 0.19 ^j^	−1.5 ± 0.02 ^i^	9.1 ± 0.20 ^h^	62.5 ± 0.11 ^h^	−2.5 ± 0.06 ^h^	6.8 ± 0.11 ^i^	NG	NG	NG
SPI-D	33	74.2 ± 0.11 ^b^	2.7 ± 0.09 ^a^	11.8 ± 0.09 ^d^	75.7 ± 0.27 ^b^	1.3 ± 0.10 ^a^	10.9 ± 0.11 ^de^	76.5 ± 0.22 ^b^	0.5 ± 0.13 ^a^	10.7 ± 0.10 ^b^
	66	73.4 ± 0.18 ^bc^	1.3 ± 0.06 ^e^	14.4 ± 0.10 ^b^	74.8 ± 0.18 ^bc^	0.6 ± 0.06 ^cd^	12.8 ± 0.13 ^b^	NG	NG	NG
	100	NG	NG	NG	NG	NG	NG	NG	NG	NG
SPC	33	73.0 ± 0.19 ^cd^	2.3 ± 0.04 ^b^	12.4 ± 0.09 ^cd^	75.6 ± 0.20 ^b^	1.0 ± 0.06 ^ab^	11.8 ± 0.11 ^c^	75.8 ± 0.17 ^b^	0.4 ± 0.05 ^a^	10.8 ± 0.19 ^b^
	66	70.7 ± 0.18 ^f^	1.6 ± 0.06 ^de^	15.4 ± 0.10 ^a^	73.4 ± 0.20 ^de^	0.4 ± 0.05 ^d^	13.3 ± 0.23 ^b^	72.6 ± 0.13 ^d^	−0.1 ± 0.04 ^b^	12.1 ± 0.11 ^a^
	100	NG	NG	NG	70.0 ± 0.10 ^f^	0.9 ± 0.04 ^bc^	14.7 ± 0.08 ^a^	NG	NG	NG

CL: control meat batter; SPI-A to -D: soy protein isolates A, B, C, and D; SPC: soy protein concentrate; NG: no gel formed. Different superscripts (^a–k^) within a column indicate significant differences (*p* < 0.05).

## Data Availability

The data presented in this study are available on reasonable request from the corresponding author.

## Supplementary Materials

The following supporting information can be downloaded at: https://www.mdpi.com/article/10.3390/foods14030427/s1, Table S1: Texture parameters (mean ± standard error) of three lean meat batter systems (0%, 40%, and 80% added water) with 33 to 100% meat replacement by soy proteins; Figure S1: Photos of soy protein powders evaluated.
