# Supplementary material for: Impact of Five Soy Proteins on Lean Chicken Breast Systems with Varying Moisture Contents: Cooking Loss, Texture, Microstructure, and T2 NMR
Source: Foods. 2025 Jan 28;14(3):427. doi: 10.3390/foods14030427 (PMC11817676; doi:10.3390/foods14030427)
Supplement: Supplementary file 1 [file foods-14-00427-s001.zip › foods-3398900-supplementary.docx]

**Supplementary Materials**

| Table S1. Texture parameters (mean ± standard error) of three lean meat batter systems (0%, 40%, and 80% added water) with 33 to 100% meat replacement by soy proteins. n=18. | | | | | | | | | | | | | | | | |
| --- | --- | --- | --- | --- | --- | --- | --- | --- | --- | --- | --- | --- | --- | --- | --- | --- |
|  | | **0% added water** | | | | | **40% added water** | | | | | **80% added water** | | | | |
| **Treatment** | **Replacement**  **(%)** | **Springiness (-)** | **Cohesiveness (-)** | **Gumminess (N)** | **Chewiness (N)** | **Resilience (-)** | **Springiness (-)** | **Cohesiveness (-)** | **Gumminess (N)** | **Chewiness (N)** | **Resilience (-)** | **Springiness (-)** | **Cohesiveness (-)** | **Gumminess (N)** | **Chewiness (N)** | **Resilience (-)** |
| CL | 0 | 0.87±0.01^f^ | 0.59±0.01^e^ | 40.5±0.67^g^ | 35.4±0.64^d^ | 0.22±0.01^h^ | 0.86±0.01^d^ | 0.48±0.01^gh^ | 8.9±0.17^f^ | 7.7±0.14^f^ | 0.14±0.01^fg^ | 0.73±0.01^e^ | 0.42±0.01^d^ | 2.7±0.11^e^ | 2.0±0.11^e^ | 0.11±0.01^d^ |
| SPI-A | 33 | 0.91±0.01^cd^ | 0.70±0.01^bc^ | 52.0±0.58^bcd^ | 47.2±0.67^b^ | 0.31±0.01^ef^ | 0.93±0.01^abc^ | 0.67±0.01^a^ | 18.5±0.20^b^ | 17.2±0.21^b^ | 0.28±0.01^ab^ | 0.90±0.01^a^ | 0.58±0.02^a^ | 7.1±0.35^b^ | 6.4±0.34^b^ | 0.21±0.01^a^ |
|  | 66 | 0.93±0.01^ab^ | 0.74±0.01^a^ | 48.2±0.75^e^ | 45.0±0.77^b^ | 0.36±0.01^abc^ | 0.91±0.01^bc^ | 0.59±0.01^cd^ | 9.6±0.19^ef^ | 8.7±0.18^ef^ | 0.23±0.01^cd^ | 0.89±0.01^abc^ | 0.53±0.01^abc^ | 3.5±0.11^de^ | 3.1±0.11^cde^ | 0.15±0.01^bc^ |
|  | 100 | 0.95±0.01^a^ | 0.70±0.01^bc^ | 14.7±0.53^h^ | 14.0±0.50^e^ | 0.36±0.01^ab^ | 0.94±0.01^ab^ | 0.51±0.01^fg^ | 2.6±0.06^h^ | 2.5±0.07^h^ | 0.16±0.01^f^ | NG | NG | NG | NG | NG |
| SPI-B | 33 | 0.91±0.01^cd^ | 0.69±0.01^cd^ | 51.3±0.52^cd^ | 46.6±0.49^b^ | 0.30±0.01^f^ | 0.91±0.01^bc^ | 0.59±0.01^cd^ | 13.8±0.31^cd^ | 12.5±0.33^cd^ | 0.23±0.01^cd^ | 0.90±0.01^ab^ | 0.59±0.02^a^ | 7.8±0.43^ab^ | 7.0±0.46^ab^ | 0.22±0.01^a^ |
|  | 66 | 0.93±0.01^bc^ | 0.72±0.01^ab^ | 44.1±0.71^f^ | 40.8±0.64^c^ | 0.33±0.01^cde^ | 0.90±0.01^c^ | 0.63±0.01^abc^ | 10.4±0.27^e^ | 9.4±0.27^e^ | 0.23±0.01^cd^ | 0.86±0.01^bcd^ | 0.56±0.01^ab^ | 4.4±0.14^cd^ | 3.8±0.13^cd^ | 0.18±0.01^ab^ |
|  | 100 | 0.93±0.01^ab^ | 0.54±0.01^f^ | 8.1±0.24^ij^ | 7.5±0.23^fg^ | 0.24±0.01^gh^ | 0.92±0.01^abc^ | 0.46±0.01^g^ | 1.6±0.04^hi^ | 1.5±0.04^hi^ | 0.09±0.01^h^ | NG | NG | NG | NG | NG |
| SPI-C | 33 | 0.90±0.01^de^ | 0.69±0.01^cd^ | 49.3±0.66^de^ | 44.4±0.66^b^ | 0.30±0.01^f^ | 0.90±0.01^cd^ | 0.60±0.01^bcd^ | 14.5±0.36^c^ | 13.1±0.38^c^ | 0.23±0.01^cd^ | 0.86±0.01^cd^ | 0.48±0.01^cd^ | 4.3±0.26^cd^ | 3.7±0.23^cd^ | 0.14±0.01^cd^ |
|  | 66 | 0.93±0.01^bc^ | 0.70±0.01^bc^ | 41.9±0.58^fg^ | 38.8±0.53^c^ | 0.32±0.01^def^ | 0.91±0.01^bc^ | 0.57±0.01^de^ | 8.8±0.14^f^ | 8.0±0.14^f^ | 0.20±0.01^de^ | 0.88±0.01^abc^ | 0.51±0.01^bc^ | 3.3±0.18^de^ | 2.9±0.17^de^ | 0.14±0.01^bcd^ |
|  | 100 | 0.94±0.01^ab^ | 0.69±0.02^bcd^ | 10.3±0.42^i^ | 9.7±0.43^f^ | 0.38±0.01^a^ | 0.95±0.01^a^ | 0.48±0.01^fg^ | 2.0±0.04^hi^ | 1.9±0.04^h^ | 0.13±0.01^g^ | NG | NG | NG | NG | NG |
| SPI-D | 33 | 0.92±0.01^bc^ | 0.72±0.01^abc^ | 54.8±0.72^ab^ | 50.6±0.73^a^ | 0.34±0.01^bcd^ | 0.92±0.01^abc^ | 0.63±0.01^abc^ | 18.1±0.53^b^ | 16.8±0.55^b^ | 0.28±0.01^a^ | 0.86±0.01^cd^ | 0.49±0.02^c^ | 4.9±0.25^c^ | 4.2±0.24^c^ | 0.18±0.01^ab^ |
|  | 66 | 0.81±0.01^g^ | 0.37±0.01^g^ | 5.8±0.15^j^ | 4.7±0.14^g^ | 0.11±0.01^i^ | 0.59±0.02^e^ | 0.38±0.01^h^ | 1.1±0.05^i^ | 0.6±0.05^i^ | 0.09±0.01^h^ | NG | NG | NG | NG | NG |
|  | 100 | NG | NG | NG | NG | NG | NG | NG | NG | NG | NG | NG | NG | NG | NG | NG |
| SPC | 33 | 0.87±0.01^f^ | 0.66±0.01^d^ | 52.9±0.72^bc^ | 45.9±0.62^b^ | 0.26±0.01^g^ | 0.91±0.01^bc^ | 0.64±0.01^ab^ | 21.3±0.33^a^ | 19.5±0.33^a^ | 0.25±0.01^bc^ | 0.89±0.01^abc^ | 0.57±0.02^a^ | 8.5±0.43^a^ | 7.6±0.42^a^ | 0.21±0.01^a^ |
|  | 66 | 0.88±0.01^ef^ | 0.66±0.01^d^ | 57.2±1.00^a^ | 50.6±1.04^a^ | 0.26±0.01^g^ | 0.90±0.01^cd^ | 0.54±0.01^ef^ | 12.7±0.28^d^ | 11.4±0.29^d^ | 0.19±0.01^e^ | 0.83±0.01^d^ | 0.53±0.01^abc^ | 4.5±0.24^cd^ | 3.8±0.24^cd^ | 0.14±0.01^bcd^ |
|  | 100 | NG | NG | NG | NG | NG | 0.93±0.01^abc^ | 0.44±0.01^g^ | 4.9±0.10^g^ | 4.6±0.11^g^ | 0.12±0.01^gh^ | NG | NG | NG | NG | NG |
| CL: control meat batter; SPI-A to D: soy protein isolates A, B, C, and D; SPC: soy protein concentrate. NG: no gel formed. Different superscripts (^a-j^) within a column indicate statistically significant differences (P < 0.05). | | | | | | | | | | | | | | | | |

| 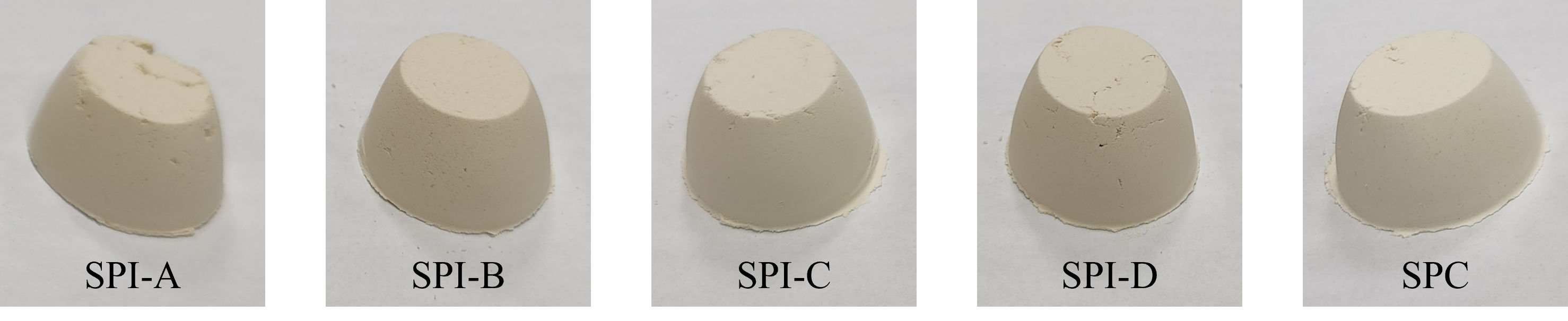 |
| --- |
| **Figure S1**. Photos of soy protein powders evaluated. SPI-A to D: soy protein isolates A, B, C, and D; SPC: soy protein concentrate. |
